# Tetra­kis(pyridine-2-carboxyl­ato-κ^2^ *N*,*O*)palladium(IV) dihydrate

**DOI:** 10.1107/S1600536809039270

**Published:** 2009-10-03

**Authors:** Nam-Ho Kim, Kwang Ha

**Affiliations:** a School of Applied Chemical Engineering, The Research Institute of Catalysis, Chonnam National University, Gwangju 500-757, Republic of Korea

## Abstract

The asymmetric unit of the title compound, [Pd(C_6_H_4_NO_2_)_4_]·2H_2_O, consists of a quarter of a neutral Pd^IV^ complex and half of a solvent water mol­ecule. In the complex, the Pd^IV^ ion is located on a fourfold inversion axis and eight-coordinated in a distorted dodeca­hedral environment by four N and four O atoms from four symmetry-related pyridine-2-carboxyl­ate (pic) anionic ligands. In the crystal, the water mol­ecule is involved in O—H⋯O hydrogen bonding, and weak inter­molecular C—H⋯O hydrogen bonds occur. There are also inter­molecular π–π inter­actions between adjacent pyridine rings, with a centroid–centroid distance of 3.715 (3) Å.

## Related literature

For details of polyhedra with coordination number eight, see: Lippard & Russ (1968 ▶); Muetterties & Guggenberger (1974 ▶). For the synthesis and structure of the Pd(II)–pic complex, [Pd(pic)_2_], see: Qin *et al.* (2002 ▶). For the crystal structures of eight-coordinated *M*(III, IV)–pic complexes (*M* = Nb, Er or Bi), see: Ooi *et al.* (1996 ▶); Soares-Santos *et al.* (2003 ▶); Callens *et al.* (2008 ▶). For the crystal structures of Pd(II) in an environment of eight O atoms, see: Izarova *et al.* (2009 ▶).
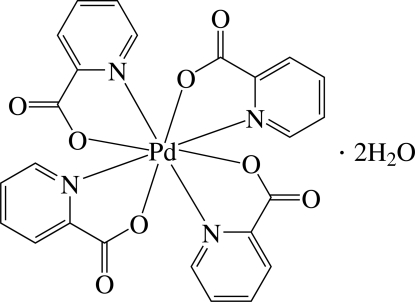


## Experimental

### 

#### Crystal data

[Pd(C_6_H_4_NO_2_)_4_]·2H_2_O
*M* *_r_* = 630.84
Tetragonal, 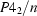

*a* = 11.1621 (5) Å
*c* = 9.5880 (9) Å
*V* = 1194.59 (14) Å^3^
*Z* = 2
Mo *K*α radiation
μ = 0.85 mm^−1^
*T* = 296 K
0.23 × 0.14 × 0.07 mm

#### Data collection

Bruker SMART 1000 CCD diffractometer
Absorption correction: multi-scan (**SADABS**; Bruker, 2000 ▶) *T*_min_ = 0.804, *T*_max_ = 0.942
8399 measured reflections
1485 independent reflections
1074 reflections with *I* > 2σ(*I*)
*R*_int_ = 0.045

#### Refinement

*R*[*F* ^2^ > 2σ(*F* ^2^)] = 0.034
*wR*(*F* ^2^) = 0.104
*S* = 1.21
1485 reflections
89 parameters
H-atom parameters constrained
Δρ_max_ = 0.76 e Å^−3^
Δρ_min_ = −1.00 e Å^−3^

### 

Data collection: *SMART* (Bruker, 2000 ▶); cell refinement: *SAINT* (Bruker, 2000 ▶); data reduction: *SAINT*; program(s) used to solve structure: *SHELXS97* (Sheldrick, 2008 ▶); program(s) used to refine structure: *SHELXL97* (Sheldrick, 2008 ▶); molecular graphics: *ORTEP-3* (Farrugia, 1997 ▶) and *PLATON* (Spek, 2009 ▶); software used to prepare material for publication: *SHELXL97*.

## Supplementary Material

Crystal structure: contains datablocks global, I. DOI: 10.1107/S1600536809039270/is2464sup1.cif

Structure factors: contains datablocks I. DOI: 10.1107/S1600536809039270/is2464Isup2.hkl

Additional supplementary materials:  crystallographic information; 3D view; checkCIF report

## Figures and Tables

**Table 1:** Hydrogen-bond geometry (Å, °)

*D*—H⋯*A*	*D*—H	H⋯*A*	*D*⋯*A*	*D*—H⋯*A*
O3—H3*O*⋯O2^i^	0.87	2.04	2.879 (5)	161
C1—H1⋯O2^ii^	0.93	2.55	3.233 (5)	131
C2—H2⋯O3^iii^	0.93	2.59	3.420 (6)	149

Symmetry codes: (i) 
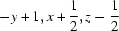
; (ii) 
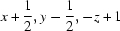
; (iii) 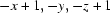.

**Fig. 1.**
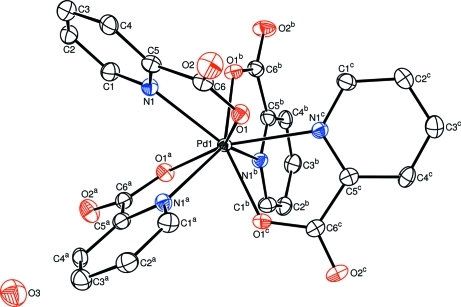
The structure of the title compound, with displacement ellipsoids drawn at the 30% probability level for non-H atoms [Symmetry codes: (a) 1/2 - x, 1/2 - y, z, (b) y, 1/2 - x, 1/2 - z, (c) 1/2 - y, x, 1/2 - z]. H atoms are omitted.

**Fig. 2.**
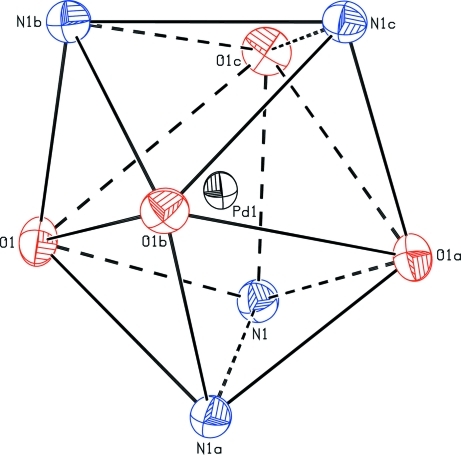
View of the distorted dodecahedral geometry around the Pd atom.

**Fig. 3.**
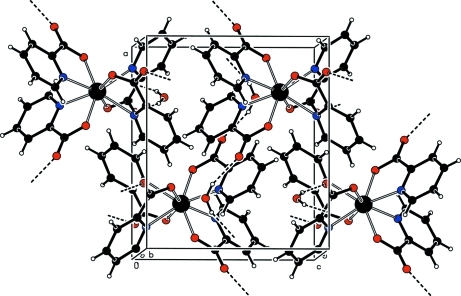
View of the unit-cell contents of the title compound. Hydrogen-bond interactions are drawn with dashed lines.

## supplementary crystallographic information

### Comment

The asymmetric unit of the title compound, [Pd(C_6_H_4_NO_2_)_4_].2H_2_O, contains a quarter of a neutral Pd^IV^ complex and one half of a solvent water molecule (Fig. 1). In the crystal, the complex has the symmetry elements *C*_2_ and *S*_4_, and its point group is *S*_4_ and the Pd atom lies on the special position at (1/4, 1/4, 1/4) (Wyckoff letter a). The water molecule is disposed about a twofold rotation axis through O atom with the special position at (3/4, 1/4, *z*) (Wyckoff letter e). In the complex, the Pd^4+^ ion is eight-coordinated in a distorted dodecahedral environment (Fig. 2) by four N and four O atoms from four distinct pyridine-2-carboxylate (pic) anionic ligands. All four ligands are coordinated in an *N*,*O*-chelation mode with the considerably different Pd—O and Pd—N bond lengths [Pd—O = 2.097 (3) Å, Pd—N = 2.373 (3) Å] and the five-membered chelate ring has an O—Pd—N bite angle of 72.07 (12)°. On the contrary, the Pd—O and Pd—N bond lengths in the Pd(II)-pic complex, [Pd(pic)_2_], are almost equal [Pd—O = 2.003 (2) Å, Pd—N = 1.998 (2) Å] and the bite angle of the chelate ring is 82.36 (9)° (Qin *et al.*, 2002). The C═O bond length [1.215 (5) Å] and the C—O bond length [1.314 (5) Å] are typical and similar to values in the complex [Pd(pic)_2_] (Qin *et al.*, 2002). In the crystal structure, the water molecule is involved in O—H···O hydrogen bonding and weak intermolecular C—H···O hydrogen bonds occur (Table 1 and Fig. 3). There are also intermolecular π-π interactions between adjacent pyridine rings, with a centroid-centroid distance of 3.715 (3) Å.

### Experimental

Single crystals of the title compound were unexpectedly obtained by reacting pyridine-2-carboxylic acid (0.721 g, 5.86 mmol), 1,6-diaminohexane (0.160 g, 1.38 mmol) and Na_2_PdCl_4_ (0.200 g, 0.68 mmol) in H_2_O (10 ml) for 24 h at room temperature. Crystals suitable for X-ray analysis were obtained by slow evaporation from a CH_3_CN solution of the white reaction product.

### Refinement

H atoms were positioned geometrically and allowed to ride on their respective parent atoms [C—H = 0.93 Å, O—H = 0.87 Å and *U*_iso_(H) = 1.2*U*_eq_(C) or 1.5*U*_eq_(O)]].

### Crystal data

**Table:**

[Pd(C_6_H_4_NO_2_)_4_]·2H_2_O	*D*_x_ = 1.754 Mg m^−^^3^
*M**_r_* = 630.84	Mo *K*α radiation, λ = 0.71073 Å
Tetragonal, *P*4_2_/*n*	Cell parameters from 3500 reflections
Hall symbol: -P 4bc	θ = 2.6–28.3°
*a* = 11.1621 (5) Å	µ = 0.85 mm^−^^1^
*c* = 9.5880 (9) Å	*T* = 296 K
*V* = 1194.59 (14) Å^3^	Block, colorless
*Z* = 2	0.23 × 0.14 × 0.07 mm
*F*(000) = 636	

### Data collection

**Table:**

Bruker SMART 1000 CCD diffractometer	1485 independent reflections
Radiation source: fine-focus sealed tube	1074 reflections with *I* > 2σ(*I*)
graphite	*R*_int_ = 0.045
φ and ω scans	θ_max_ = 28.3°, θ_min_ = 2.6°
Absorption correction: multi-scan (*SADABS*; Bruker, 2000)	*h* = −14→14
*T*_min_ = 0.804, *T*_max_ = 0.942	*k* = −14→10
8399 measured reflections	*l* = −12→12

### Refinement

**Table:**

Refinement on *F*^2^	Primary atom site location: structure-invariant direct methods
Least-squares matrix: full	Secondary atom site location: difference Fourier map
*R*[*F*^2^ > 2σ(*F*^2^)] = 0.034	Hydrogen site location: inferred from neighbouring sites
*wR*(*F*^2^) = 0.104	H-atom parameters constrained
*S* = 1.21	*w* = 1/[σ^2^(*F*_o_^2^) + (0.031*P*)^2^ + 2.1364*P*] where *P* = (*F*_o_^2^ + 2*F*_c_^2^)/3
1485 reflections	(Δ/σ)_max_ < 0.001
89 parameters	Δρ_max_ = 0.76 e Å^−^^3^
0 restraints	Δρ_min_ = −1.00 e Å^−^^3^

### Special details

**Table:**

Geometry. All e.s.d.'s (except the e.s.d. in the dihedral angle between two l.s. planes) are estimated using the full covariance matrix. The cell e.s.d.'s are taken into account individually in the estimation of e.s.d.'s in distances, angles and torsion angles; correlations between e.s.d.'s in cell parameters are only used when they are defined by crystal symmetry. An approximate (isotropic) treatment of cell e.s.d.'s is used for estimating e.s.d.'s involving l.s. planes.
Refinement. Refinement of *F*^2^ against ALL reflections. The weighted *R*-factor *wR* and goodness of fit *S* are based on *F*^2^, conventional *R*-factors *R* are based on *F*, with *F* set to zero for negative *F*^2^. The threshold expression of *F*^2^ > σ(*F*^2^) is used only for calculating *R*-factors(gt) *etc*. and is not relevant to the choice of reflections for refinement. *R*-factors based on *F*^2^ are statistically about twice as large as those based on *F*, and *R*- factors based on ALL data will be even larger.

### Fractional atomic coordinates and isotropic or equivalent isotropic displacement parameters (Å^2^)

**Table:**

	*x*	*y*	*z*	*U*_iso_*/*U*_eq_	
Pd1	0.2500	0.2500	0.2500	0.02277 (17)	
O1	0.0856 (2)	0.3186 (3)	0.3193 (3)	0.0380 (7)	
O2	−0.0577 (3)	0.3299 (3)	0.4796 (4)	0.0570 (10)	
N1	0.1892 (3)	0.1367 (3)	0.4471 (4)	0.0334 (8)	
C1	0.2408 (4)	0.0371 (4)	0.4990 (5)	0.0416 (10)	
H1	0.3108	0.0087	0.4581	0.050*	
C2	0.1927 (4)	−0.0243 (4)	0.6114 (5)	0.0449 (11)	
H2	0.2298	−0.0930	0.6454	0.054*	
C3	0.0889 (4)	0.0180 (5)	0.6722 (5)	0.0491 (12)	
H3	0.0561	−0.0210	0.7489	0.059*	
C4	0.0343 (4)	0.1187 (4)	0.6182 (4)	0.0406 (10)	
H4	−0.0360	0.1482	0.6574	0.049*	
C5	0.0863 (4)	0.1755 (4)	0.5038 (5)	0.0366 (10)	
C6	0.0317 (4)	0.2821 (4)	0.4335 (5)	0.0371 (9)	
O3	0.7500	0.2500	0.1567 (7)	0.092 (2)	
H3O	0.7183	0.3148	0.1215	0.137*	

### Atomic displacement parameters (Å^2^)

**Table:**

	*U*^11^	*U*^22^	*U*^33^	*U*^12^	*U*^13^	*U*^23^
Pd1	0.02072 (19)	0.02072 (19)	0.0269 (3)	0.000	0.000	0.000
O1	0.0315 (15)	0.0398 (17)	0.0426 (17)	0.0077 (12)	−0.0017 (14)	0.0034 (14)
O2	0.0388 (19)	0.066 (2)	0.066 (2)	0.0183 (16)	0.0149 (17)	0.0011 (19)
N1	0.0312 (18)	0.0364 (19)	0.0326 (18)	0.0020 (14)	0.0007 (15)	0.0005 (15)
C1	0.040 (3)	0.035 (2)	0.049 (3)	0.0050 (18)	0.000 (2)	0.003 (2)
C2	0.053 (3)	0.040 (3)	0.042 (3)	−0.004 (2)	−0.007 (2)	0.006 (2)
C3	0.052 (3)	0.056 (3)	0.040 (3)	−0.006 (2)	0.001 (2)	0.006 (2)
C4	0.039 (2)	0.056 (3)	0.027 (2)	−0.004 (2)	0.0044 (18)	−0.006 (2)
C5	0.035 (2)	0.034 (2)	0.041 (2)	−0.0023 (17)	−0.0010 (18)	−0.0059 (19)
C6	0.032 (2)	0.038 (2)	0.041 (2)	−0.0005 (18)	0.0022 (19)	−0.006 (2)
O3	0.121 (6)	0.064 (4)	0.090 (5)	0.002 (4)	0.000	0.000

### Geometric parameters (Å, °)

**Table:**

Pd1—O1^i^	2.097 (3)	N1—C1	1.347 (5)
Pd1—O1^ii^	2.097 (3)	C1—C2	1.386 (6)
Pd1—O1^iii^	2.097 (3)	C1—H1	0.9300
Pd1—O1	2.097 (3)	C2—C3	1.380 (7)
Pd1—N1^ii^	2.373 (3)	C2—H2	0.9300
Pd1—N1^iii^	2.373 (3)	C3—C4	1.380 (7)
Pd1—N1^i^	2.373 (3)	C3—H3	0.9300
Pd1—N1	2.373 (3)	C4—C5	1.393 (6)
O1—C6	1.314 (5)	C4—H4	0.9300
O2—C6	1.215 (5)	C5—C6	1.497 (6)
N1—C5	1.343 (5)	O3—H3O	0.87
			
O1^i^—Pd1—O1^ii^	95.77 (5)	N1^ii^—Pd1—N1	74.42 (17)
O1^i^—Pd1—O1^iii^	143.04 (17)	N1^iii^—Pd1—N1	129.37 (10)
O1^ii^—Pd1—O1^iii^	95.77 (5)	N1^i^—Pd1—N1	129.37 (10)
O1^i^—Pd1—O1	95.77 (5)	C6—O1—Pd1	123.4 (3)
O1^ii^—Pd1—O1	143.04 (17)	C5—N1—C1	118.8 (4)
O1^iii^—Pd1—O1	95.77 (5)	C5—N1—Pd1	113.3 (3)
O1^i^—Pd1—N1^ii^	71.43 (12)	C1—N1—Pd1	127.7 (3)
O1^ii^—Pd1—N1^ii^	72.07 (12)	N1—C1—C2	122.0 (4)
O1^iii^—Pd1—N1^ii^	145.42 (12)	N1—C1—H1	119.0
O1—Pd1—N1^ii^	78.64 (12)	C2—C1—H1	119.0
O1^i^—Pd1—N1^iii^	78.64 (12)	C3—C2—C1	118.9 (4)
O1^ii^—Pd1—N1^iii^	71.43 (12)	C3—C2—H2	120.5
O1^iii^—Pd1—N1^iii^	72.07 (12)	C1—C2—H2	120.5
O1—Pd1—N1^iii^	145.42 (12)	C2—C3—C4	119.5 (5)
N1^ii^—Pd1—N1^iii^	129.37 (10)	C2—C3—H3	120.3
O1^i^—Pd1—N1^i^	72.07 (12)	C4—C3—H3	120.3
O1^ii^—Pd1—N1^i^	145.42 (12)	C3—C4—C5	118.8 (4)
O1^iii^—Pd1—N1^i^	78.64 (12)	C3—C4—H4	120.6
O1—Pd1—N1^i^	71.43 (12)	C5—C4—H4	120.6
N1^ii^—Pd1—N1^i^	129.37 (10)	N1—C5—C4	121.9 (4)
N1^iii^—Pd1—N1^i^	74.42 (17)	N1—C5—C6	115.0 (4)
O1^i^—Pd1—N1	145.42 (12)	C4—C5—C6	123.1 (4)
O1^ii^—Pd1—N1	78.64 (12)	O2—C6—O1	122.9 (4)
O1^iii^—Pd1—N1	71.43 (12)	O2—C6—C5	121.3 (4)
O1—Pd1—N1	72.07 (12)	O1—C6—C5	115.8 (4)
			
O1^i^—Pd1—O1—C6	−141.3 (3)	N1^iii^—Pd1—N1—C1	24.3 (4)
O1^ii^—Pd1—O1—C6	−33.7 (3)	N1^i^—Pd1—N1—C1	127.2 (4)
O1^iii^—Pd1—O1—C6	73.9 (3)	C5—N1—C1—C2	−1.9 (7)
N1^ii^—Pd1—O1—C6	−71.6 (3)	Pd1—N1—C1—C2	−176.7 (3)
N1^iii^—Pd1—O1—C6	140.4 (3)	N1—C1—C2—C3	−0.1 (7)
N1^i^—Pd1—O1—C6	149.9 (3)	C1—C2—C3—C4	1.3 (7)
N1—Pd1—O1—C6	5.6 (3)	C2—C3—C4—C5	−0.4 (7)
O1^i^—Pd1—N1—C5	71.4 (4)	C1—N1—C5—C4	2.8 (6)
O1^ii^—Pd1—N1—C5	155.0 (3)	Pd1—N1—C5—C4	178.3 (3)
O1^iii^—Pd1—N1—C5	−104.9 (3)	C1—N1—C5—C6	−176.3 (4)
O1—Pd1—N1—C5	−2.2 (3)	Pd1—N1—C5—C6	−0.7 (4)
N1^ii^—Pd1—N1—C5	80.7 (3)	C3—C4—C5—N1	−1.6 (7)
N1^iii^—Pd1—N1—C5	−150.8 (3)	C3—C4—C5—C6	177.4 (4)
N1^i^—Pd1—N1—C5	−47.9 (3)	Pd1—O1—C6—O2	173.0 (3)
O1^i^—Pd1—N1—C1	−113.6 (4)	Pd1—O1—C6—C5	−7.9 (5)
O1^ii^—Pd1—N1—C1	−29.9 (4)	N1—C5—C6—O2	−175.7 (4)
O1^iii^—Pd1—N1—C1	70.2 (4)	C4—C5—C6—O2	5.2 (7)
O1—Pd1—N1—C1	172.9 (4)	N1—C5—C6—O1	5.1 (6)
N1^ii^—Pd1—N1—C1	−104.3 (4)	C4—C5—C6—O1	−173.9 (4)

Symmetry codes: (i) *y*, −*x*+1/2, −*z*+1/2; (ii) −*x*+1/2, −*y*+1/2, *z*; (iii) −*y*+1/2, *x*, −*z*+1/2.

### Hydrogen-bond geometry (Å, °)

**Table:**

*D*—H···*A*	*D*—H	H···*A*	*D*···*A*	*D*—H···*A*
O3—H3O···O2^iv^	0.87	2.04	2.879 (5)	161
C1—H1···O2^v^	0.93	2.55	3.233 (5)	131
C2—H2···O3^vi^	0.93	2.59	3.420 (6)	149

Symmetry codes: (iv) −*y*+1, *x*+1/2, *z*−1/2; (v) *x*+1/2, *y*−1/2, −*z*+1; (vi) −*x*+1, −*y*, −*z*+1.
